# Hematopoietic stem cell transplantation for multiple sclerosis: no inflammation, no response

**DOI:** 10.1111/ene.16565

**Published:** 2024-12-18

**Authors:** Richard K. Burt, Tobias Alexander, the EBMT Autoimmune Diseases Working Party (ADWP)

**Affiliations:** ^1^ Northwestern University Chicago Illinois USA; ^2^ Scripps hematology La Jolla California USA; ^3^ Genani Corporation Chicago Illinois USA; ^4^ Department of Rheumatology and Clinical Immunology Charité—Universitätsmedizin Berlin Berlin Germany; ^5^ EBMT Autoimmune Disease Working Party (EBMT) Berlin Germany

In this edition of the *European Journal of Neurology*, Braun et al. report results from a systematic literature review of publications reporting on autologous hematopoietic stem cell transplantation (HSCT) for multiple sclerosis (MS) undergoing progressive neurological decline in baseline without clinical relapses or decline between clinical relapses. They compared 665 patients with progressive multiple sclerosis (PMS) of which 74 were primary progressive MS (PPMS) defined as PMS from diagnosis and 591 with secondary progressive MS (SPMS) defined as PMS after an initial relapsing–remitting course. As controls they used 647 patients with RRMS defined as a relapsing–remitting course with stable neurological disability between relapses [1].

There are 16 disease modifying therapies (DMTs) approved by the Food and Drug Administration for RRMS. The effectiveness of DMTs for PMS is questionable or at best less clear. Multiple studies have demonstrated superiority of HSCT for RRMS including a large meta‐analysis of 4831 patients [2] and a phase III randomized trial [3]. Because an effective therapy is needed for PMS, Braun et al. summarized the published outcome of HSCT for PMS. They found highly variable results in PMS patients in terms of achieving progression‐free survival and no evidence of disease activity (NEDA‐3), suggesting that HSCT does not halt progression in this patient cohort but may provide clinical benefit in selected patients.

RRMS is a dynamic and repetitive immune mediated breakdown in the blood–brain barrier, extravasation of immune cells, and macrophage stripping of myelin followed healing of the blood–brain barrier, remyelination and repair [4]. Neuronal damage starts during RRMS and becomes the predominate pathophysiology during SPMS [5]. PPMS is predominately neuronal degenerative from onset [6]. For this reason, immune based therapies including all DMTs and autologous HSCT are most effective in RRMS but of limited value for PMS (PPMS or SPMS) [6]. During the transition from predominately inflammatory RRMS into late SPMS, patients transition through a stage of SPMS with residual smoldering inflammation [7]. It is during this stage that immune based therapies such as HSCT are still of benefit, albeit to a lesser amount. On this background, recent guidelines and recommendations from the European Group for Blood and Marrow Transplantation (EBMT) Autoimmune Diseases Working Party (ADWP) and the Joint Accreditation Committee of EBMT and the International Society for Cellular Therapy (ISCT) (JACIE) suggest HSCT as standard indication in patients with active relapsing–remitting MS failing DMTs, and a clinical option in patients with progressive MS with active inflammatory component, but generally not in patients with progressive MS without active inflammatory component [8].

Identifying subsets of PMS that may still benefit from HSCT is essential. Two such subsets are active SPMS and potentially those with progression independent of relapse activity (PIRA). SPMS may be divided into active SPMS and non‐active SPMS [9]. Non‐active SPMS is quiescent in terms of relapses and new magnetic resonance imaging (MRI) lesions [8]. Active SPMS manifests a clinical relapse or a new MRI lesion over the prior year [8]. Using a non‐myeloablative HSCT regimen of cyclophosphamide and anti‐thymocyte globulin (ATG), the neurological disability of patients with active SPMS improved [10]. RRMS and active SPMS had a sustained mean decline in Expanded Disability Status Scale of >1.0 and >0.5 points, respectively, after HSCT [9]. Non‐active SPMS did not improve [10]. The extent to which active neuronal inflammation at baseline had an influence on the reported results of Braun et al. in PMS patients remains unclear, as no data on this subject were included. Nevertheless, the available data demonstrating that PPMS patients benefited less than SPMS and particularly RRMS patients clearly indicate that the less inflammation the lower the response to HSCT.

Patients treated with anti‐B cell monotherapy may have slow silent accumulation of disability without clinical relapses or MRI changes, a phenomenon termed PIRA [11]. To ensure that MRI imaging was included in truly defining PIRA, recently the term PIRMA (progression independent of relapse and MRI activity) was introduced [12]. Relapse‐independent accumulation of disability has been documented for patients on natalizumab [13]. Recently more specialized MRI analysis has documented persistent paramagnetic rim lesions in patients on ocrelizumab [14]. Such studies suggest that PIRMA arises from incomplete treatment of smoldering inflammation not evident by gross clinical relapse or on routine MRI. MRI paramagnetic rim lesions are associated with PIRMA and arise from and are consistent with persistent low‐grade inflammation, free radical generation, and iron deposition in activated microglia and immune cells located on the periphery of chronic active lesions.

Why is PIRMA so prevalent for patients on anti‐CD20 B cell monoclonal therapy? B cells play a role in MS and traffic into and out of acute lesions and are only a small percentage of immune cells within chronic active lesions [15]. It is therefore possible that B cell directed therapies do not cover smoldering low‐grade T cell mediated inflammation. Elimination of B cell activity but unchecked T cell reactivity may lead to no noticeable clinical attacks and no new MRI lesions on routine MRI but slow progression of disability, that is, PIRMA. Recently we have performed HSCT with a non‐myeloablative regimen using cyclophosphamide and ATG in several patients on anti‐CD20 monoclonal antibody therapy with PIRMA. All had improvement in Expanded Disability Status Scale and reversal of symptoms after HSCT (unpublished), suggesting that the mechanisms of HSCT would go beyond a single cell line effect (T or B cell directed).

In conclusion, the report by Braun et al. confirms previous findings demonstrating better outcomes in RRMS compared to SPMS and PPMS patients and suggests that central nervous system inflammation is a predictor for response. While HSCT is usually ineffective in late SPMS (non‐active SPMS) or PPMS, there is early evidence that some types of PMS such as active SPMS and PIRMA while on anti‐CD20 B cell therapy do benefit from HSCT probably because of continued smoldering inflammation. Further studies are required in MS to clearly demonstrate the efficacy of HSCT in reducing PIRMA, and these investigations should use harmonized definitions of PIRMA [16]. These aspects should be considered when selecting patients for HSCT in addition to failure of available DMTs.

## AUTHOR CONTRIBUTIONS

**Richard K. Burt:** Writing – original draft. **Tobias Alexander:** Writing – original draft.

## CONFLICT OF INTEREST STATEMENT

The authors declared no conflict of interest.

## Data Availability

Data sharing is not applicable to this article as no new data were created or analyzed in this study.
